# Effectiveness of B/F/TAF in adults with HIV who are viremic with M184V/I

**DOI:** 10.1186/s12981-025-00795-9

**Published:** 2025-11-28

**Authors:** Charlotte-Paige Rolle, Michelle L. D’Antoni, Roberto Corales, Andrea Marongiu, Joshua Gruber, Tanya Schreibman, Dionne Bell, Chiu-Bin Hsiao, Indira Brar

**Affiliations:** 1 Orlando Immunology Center, Orlando, FL USA; 2 Gilead Sciences, Inc., 333 Lakeside Drive, Foster City, CA 94404 USA; 3 Gilead Sciences Europe Ltd, Uxbridge, UK; 4 CAN Community Health, Sarasota, FL USA; 5 Capitol City Family Health Center, Baton Rouge, LA USA; 6 Positive Health Clinic, Allegheny Health Network, Pittsburgh, PA USA; 7 Henry Ford Health System, Detroit, MI USA

**Keywords:** Effectiveness of B/F/TAF, Viremia, M184V/I

## Abstract

Bictegravir/emtricitabine/tenofovir alafenamide (B/F/TAF) is indicated for people with HIV who are virologically suppressed, including those with M184V/I. Data on B/F/TAF effectiveness during viremia with M184V/I are limited. This observational study retrospectively collected clinical/demographic data from adults with viremia and M184V/I receiving B/F/TAF or alternate antiretroviral therapy (ART). Virologic suppression at 3 and ≥ 6 months was evaluated. For participants with data, 5/5 (100%) and 7/8 (88%) on B/F/TAF and 4/6 (67%) and 7/10 (70%) on alternate ART achieved virologic suppression at 3 and ≥ 6 months, respectively. Virologic suppression was achieved in most people with HIV who were viremic with M184V/I on B/F/TAF, as with alternate ART.

## Introduction

Pre-existing drug resistance can affect antiretroviral therapy (ART) effectiveness [1]. M184V and/or M184I (M184V/I) are resistance-associated mutations (RAMs) in reverse transcriptase (RT) that can be transmitted, or that emerge when emtricitabine (FTC)- and lamivudine (3TC)-containing regimens fail (in up to ~ 70% of individuals) [2–4]. With FTC and 3TC being components of most recommended regimens for decades, M184V/I reporting during routine clinical resistance testing in the US is high (2018–2024: 8–12% plasma RNA; 12–19% proviral DNA samples) [5].

The single-tablet regimen bictegravir/emtricitabine/tenofovir alafenamide (B/F/TAF) has demonstrated efficacy/effectiveness, safety, and a high barrier to resistance in clinical trials and routine clinical practice [6–8]. This efficacy extends to virologically suppressed (HIV-1 RNA < 50 copies/mL) study participants with pre-existing M184V/I [9]. Accordingly, B/F/TAF is now indicated to replace current ART in those who are virologically suppressed with no RAMs to bictegravir or tenofovir [10]. Data on whether B/F/TAF is effective in people with HIV who have viremia with M184V/I are limited. In a real-world case series examining the effectiveness of B/F/TAF in the presence of M184V/I, 4 of 6 people with HIV who were viremic at baseline achieved suppression after 12 months; the 2 unsuppressed individuals had substantial adherence lapses and 1 had an opportunistic infection [11]. In phase 3 B/F/TAF clinical trials, there are rare cases of participants with detectable viral loads and documented M184V/I who went on to achieve virologic suppression for ≥ 57 weeks on B/F/TAF [12, 13]. Additional data are needed to understand treatment options for people with HIV who are viremic with M184V/I.

The objective here was to investigate virologic suppression among people with HIV who had viremia with documented M184V/I and were treated with B/F/TAF alone or an alternate ART.

## Methods

### Study design and participants

This retrospective, multicenter, observational study collected de-identified demographic, clinical, and laboratory data from electronic medical records from 5 US clinics. Sites provided case report forms for adults with HIV (≥ 18 years) treated with B/F/TAF or alternate ART (to contextualize results and capture current treatment landscape) after February 7, 2018. Eligibility criteria included viremia (HIV-1 RNA ≥ 400 copies/mL), documentation of M184V/I at the time of viremia, and availability of ≥ 6 months of post-treatment initiation data. Participants were treatment naïve or treatment experienced, or individuals who seroconverted while on HIV pre-exposure prophylaxis (PrEP).

Allowable resistance included  ≤ 2 thymidine analog mutations (TAMs), or non-nucleoside reverse transcriptase inhibitor (NNRTI) or protease inhibitor (PI) RAMs. Pre-existing RAMs conferring resistance to the components of B/F/TAF (with the exception of M184V/I), including integrase strand transfer inhibitor (INSTI) RAMs, or ≥ 3 TAMs and K65R in RT, were exclusionary. Participants with opportunistic infections that prevented ART administration were considered ineligible.

### Assessments, outcomes, and statistical analysis

Pre-existing RAMs in protease (PR), RT, and integrase (IN) documented from commercial or local genotyping assays were tabulated. The proportion of participants achieving virologic suppression (HIV-1 RNA < 50 copies/mL) was determined after 3 and ≥ 6 months of treatment on B/F/TAF (B/F/TAF cohort) or alternate ART (alternate ART cohort). Adherence reported by healthcare providers was categorically defined as a percentage of missed doses. Data were summarized using univariate descriptive statistics.

## Results

### Baseline demographic and clinical characteristics

In total, 37 participants were screened; of these, 18 met study eligibility criteria and were enrolled. Of the 18 participants with viremia and M184V/I, 8 received B/F/TAF alone and 10 received alternate ART. Participants were predominantly male and Black, with a baseline median (quartile [Q]1–Q3) viral load of 14 227 (4370–72 800) copies/mL and a baseline median (Q1–Q3) CD4 count of 359 (200–613) cells/µL (*n* = 16; Table 1). One participant (B/F/TAF) was treatment naïve; the remaining participants were treatment experienced, 5 of whom had a history of HIV PrEP use.

**Table 1:** Baseline demographic and clinical characteristics

Characteristic, *n* (%)^a^		B/F/TAF cohort (*n* = 8)	Alternate ART cohort (*n* = 10)	Total (*N* = 18)
Age, median (Q1–Q3), years		47 (39–54)	58 (50–61)	53 (44–61)
Male sex at birth		4 (50)	7 (70)	11 (61)
Race				
Black		4 (50)	7 (70)	11 (61)
White		3 (38)	3 (30)	6 (33)
Asian		1 (13)	0 (0)	1 (6)
Viral load, median (Q1–Q3), copies/mL		11112 (1725–68 100)	17752 (6429–93 400)	14227 (4370–72 800)
CD4 cell count, median (Q1–Q3), cells/µL		503 (262–661) (*n* = 6)	303 (189–565) (*n* = 10)	359 (200–613) (*n* = 16)
CD4 cell nadir, median (Q1–Q3), cells/µL		380 (172–689)	269 (100–797)	325 (136–695)
Other baseline resistance substitutions^b^				
NNRTI		4 (50)	6 (60)	10 (56)
PI		2 (25)	1 (10)	3 (17)
Additional NRTI		0	2 (20)	2 (11)
INSTI		0	2 (20)	2 (11)
Participants with multiclass drug resistance^c^		4 (50)	7 (70)	11 (61)
Number of current ART, median (Q1–Q3)		3 (3–3)	5 (3–5)	3 (3–5)

ART, antiretroviral therapy; B/F/TAF, bictegravir/emtricitabine/tenofovir alafenamide; IAS-USA, International Antiviral Society–USA; IN, integrase; INSTI, integrase strand transfer inhibitor; NNRTI, non-nucleoside reverse transcriptase inhibitor; NRTI, nucleos(t)ide reverse transcriptase inhibitor; PI, protease inhibitor; PR, protease; Q, quartile; R, resistance; RAM, resistance-associated mutation; RT, reverse transcriptase; TAM, thymidine analog mutation

The baseline visit window was defined as up to 30 days before and 1 day after initiation of B/F/TAF or alternate ART.

^a^Unless otherwise specified. ^b^HIV-1 drug RAMs are based on IAS-USA list [14]: Primary NNRTI-R substitutions were L100I, K101E/P, K103N/S, V106A/M, V108I, E138A/G/K/Q/R, V179L, Y181C/I/V, Y188C/H/L, G190A/E/Q/S, H221Y, P225H, F227C, and M230I/L in RT. Primary PI-R substitutions were D30N, V32I, M46I/L, I47A/V, G48V, I50L/V, I54M/L, Q58E, T74P, L76V, V82A/F/L/S/T, N83D, I84V, N88S, and L90M in PR. Primary NRTI-R substitutions were K65R/E/N, T69 insertions, K70E, L74V/I, Y115F, Q151M, M184V/I, and TAMs (M41L, D67N, K70R, L210W, T215F/Y, K219E/N/Q/R) in RT. Primary INSTI-R substitutions were T66I/A/K, E92Q/G, F121Y, Y143R/H/C, S147G, Q148H/K/R, N155H/S, and R263K in IN. ^c^Multiclass resistance is defined as resistance to ≥ 2 drug classes.

Regimens in the alternate ART cohort included quadruple therapy (*n* = 4), comprising B/F/TAF with a fourth agent (darunavir boosted with cobicistat [DRV/c; *n* = 2] or doravirine [*n* = 1], and dolutegravir [DTG] + DRV/c/F/TAF [*n* = 1]); triple therapy (DTG + F/TAF [*n* = 2], DRV/c/F/TAF [*n* = 1], and DTG/rilpivirine + DRV/c [*n* = 1]); dual therapy (DTG + atazanavir [ATV; *n* = 1]); or monotherapy (DRV [*n* = 1]).

Genotypic PR and RT data were available for 18 participants and IN data for 11 participants. In addition to the M184V/I nucleos(t)ide reverse transcriptase inhibitor (NRTI) RAM present in all participants, the following number of participants had additional documented primary RAMs: NRTI: *n* = 2, NNRTI: *n* = 10, PI: *n* = 3, and INSTI: *n* = 2 (Table 1). In the B/F/TAF cohort, excluding M184V/I, no primary NRTI RAMs were reported; primary NNRTI RAMs were reported in 4 participants (K101E [*n* = 1], E138K [*n* = 2], G190A [*n* = 1], M230I [*n* = 2]), primary PI RAMs were reported in 2 participants (D30N [*n* = 1], L90M [*n* = 1]), and no INSTI resistance was reported. In the alternate ART cohort, in addition to M184V/I, primary NRTI RAMs were reported in 2 participants (D67N [*n* = 1], K70E [*n* = 1], L74V [*n* = 1]), primary NNRTI RAMs were reported in 6 participants (K101P [*n* = 1], K103N [*n* = 2], E138A [*n* = 1], Y181C [*n* = 1], Y188L [*n* = 1], G190A [*n* = 1], M230I [*n* = 2]), primary PI RAMs were reported in 1 participant (D30N [*n* = 1], M46I [*n* = 1]), and primary INSTI RAMs were reported in 2 participants (T66I [*n* = 1], E92Q [*n* = 1], T97A [*n* = 1]). Other than M184V/I, no RAMs conferred loss of susceptibility to other components of the regimen.tk 1

For participants on B/F/TAF, 4/8 (50%) had 1-drug class resistance (NRTI), 2/8 (25%) had 2-drug class resistance (NRTI + NNRTI), and 2/8 (25%) had 3-drug class resistance (NRTI + NNRTI + PI). In participants on alternate ART, 3/10 (30%) had 1-drug class resistance (NRTI), 5/10 (50%) had 2-drug class resistance (NRTI + NNRTI [*n* = 4], NRTI + INSTI [*n* = 1]), and 2/10 (20%) had 3-drug class resistance (NRTI + NNRTI + PI [*n* = 1], NRTI + NNRTI + INSTI [*n* = 1]).

### Effectiveness and postbaseline data

**Fig. 1:**
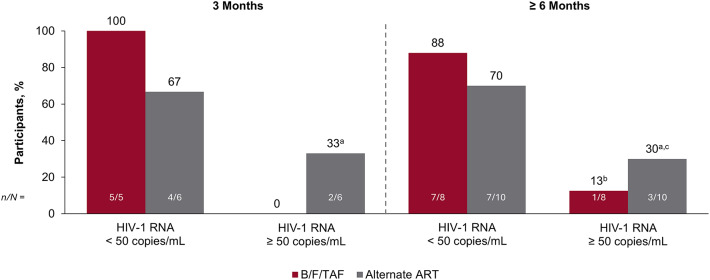
Virologic outcomes at 3 and ≥ 6 months after treatment initiation/switch. Only participants with available data are shown. ART, antiretroviral therapy; ATV, atazanavir; B/F/TAF, bictegravir/emtricitabine/tenofovir alafenamide; c, cobicistat; DRV, darunavir; DTG, dolutegravir. ^a^One participant on DTG + ATV with 75–84% adherence had viral load 7830 copies/mL at 3 months and subsequently resuppressed at 6 months (viral load 30 copies/mL); 1 participant on DRV with 100% adherence had viral load 51 copies/mL at 3 months and 913 copies/mL at 14 months (≥ 6-month timepoint). ^b^One participant on B/F/TAF with 75–84% adherence had no data at 3 months and viral load 328 copies/mL at 6 months; this participant discontinued treatment due to reported lack of efficacy and subsequently achieved virologic suppression on DRV + DTG. ^c^One participant on DTG + cDRV/F/TAF with 50–74% adherence had no data at 3 months and viral load 625 copies/mL at 6 months; 1 participant on cDRV/F/TAF with a reported adherence range of 0–24% was virologically suppressed at 3 months and had viral load 6642 copies/mL at 6 months

Among participants with data available, 5/5 (100%) and 7/8 (88%) in the B/F/TAF cohort and 4/6 (67%) and 7/10 (70%) in the alternate ART cohort had virologic suppression at 3 and ≥ 6 months, respectively (Fig. 1). For participants who had HIV-1 RNA ≥ 50 copies/mL at 3 and/or ≥ 6 months, the median (Q1–Q3) of the highest viral load at these timepoints for each participant was 913 (625–6642) copies/mL (*n* = 5) and was generally associated with suboptimal adherence. No postbaseline resistance data were available. CD4 counts were relatively stable throughout the study (data not shown). Median (Q1–Q3) treatment duration was 99 (48–169) weeks with B/F/TAF alone and 149 (36–170) weeks with alternate ART.

## Discussion

This observational retrospective analysis sought to document the impact of RAMs on the effectiveness of ART, specifically in people with HIV who had viremia with documented M184V/I and were receiving B/F/TAF.

Virologic suppression rates were high for participants on B/F/TAF (88%) and alternate ART (70%) after at least 6 months of follow-up. Our findings for B/F/TAF are in line with results for DTG + 2 NRTI regimens from the Nucleosides And Darunavir/Dolutegravir In Africa (NADIA) study, where high virologic suppression was demonstrated in participants receiving DTG + tenofovir disoproxil fumarate + 3TC-containing regimens as a second-line therapy, even when M184V/I was present at baseline [15]. B/F/TAF effectiveness in people with HIV who had viremia in the presence of M184V/I may be linked to several factors, including the high barrier to resistance [16] and long dissociation half-life of bictegravir [17], the residual antiviral activity of 3TC [18], and the impact of M184V/I on viral fitness [19] and hypersensitivity to tenofovir [20].

While treatment of HIV with B/F/TAF among people with HIV who are viremic with M184V/I is off-label, the diversity and complexity of regimens from the alternate ART cohort reflect a potential need for simple and effective options. For instance, individuals on multi-tablet regimens may benefit from the advantageous properties of potent, effective, and well-tolerated single-tablet regimens, avoiding issues such as selective adherence. Moreover, in the presence of M184V/I, treatment options can be limited as many NRTI backbones include FTC or 3TC. Additionally, in approximately two-thirds of participants, M184V/I was documented along with drug resistance in other classes, potentially further restricting regimen options. Of note, at the time the study was conducted, long-acting agents were not available.

Limitations include a retrospective study design and small sample size, precluding a formal comparison between cohorts. Many screened participants had historical M184V/I but did not have current genotype reports available during viremia, and thus the presence of M184V/I could not be confirmed. However, RAMs can be archived and potentially reactivated; therefore, M184V/I could have been circulating at the time of viremia, underscoring the importance of considering all previous resistance testing as recommended in treatment guidelines. This is a likely scenario that occurs in routine clinical practice and this population should also be studied. Additionally, postbaseline genotype data were not available. This information would be particularly useful for participants who did not achieve virologic suppression at 6 months (1/8 participants on B/F/TAF and 3/10 participants on alternate ART). This warrants further investigation in future studies.

In conclusion, virologic suppression was achieved in most people with HIV who were viremic with M184V/I treated with B/F/TAF; virologic suppression rates were comparable to alternate, often more complex, ART regimens. While a small dataset, these results expand current experience with B/F/TAF in this context.

## Data Availability

Gilead Sciences shares anonymized individual patient data upon request or as required by law or regulation with qualified external researchers based on submitted curriculum vitae and reflecting non-conflict of interest. The request proposal must also include a statistician. Approval of such requests is at Gilead Science’s discretion and is dependent on the nature of the request, the merit of the research proposed, the availability of the data, and the intended use of the data. Data requests should be sent to datarequest@gilead.com.

## Abbreviations

3TC: Lamivudine
ART: Antiretroviral therapy
ATV: Atazanavir
B/F/TAF: Bictegravir/emtricitabine/tenofovir alafenamide
c: Cobicistat
DRV: Darunavir
DTG: Dolutegravir
FTC: Emtricitabine
IN: Integrase
INSTI: Integrase strand transfer inhibitor
NADIA: Nucleosides And Darunavir/Dolutegravir In Africa
NNRTI: Non-nucleoside reverse transcriptase inhibitor
NRTI: Nucleos(t)ide reverse transcriptase inhibitor
PI: Protease inhibitor
PR: Protease
PrEP: Pre-exposure prophylaxis
Q: Quartile
RAM: Resistance-associated mutation
RT: Reverse transcriptase
TAM: Thymidine analog mutation
